# Activin A-Smad Signaling Mediates Connective Tissue Growth Factor Synthesis in Liver Progenitor Cells

**DOI:** 10.3390/ijms17030408

**Published:** 2016-03-22

**Authors:** Ze-Yang Ding, Guan-Nan Jin, Wei Wang, Yi-Min Sun, Wei-Xun Chen, Lin Chen, Hui-Fang Liang, Pran K. Datta, Ming-Zhi Zhang, Bixiang Zhang, Xiao-Ping Chen

**Affiliations:** 1 Hepatic Surgery Center, Tongji Hospital, Tongji Medical College, Huazhong University of Science and Technology, Wuhan 430030, China; dingzeyang@hust.edu.cn (Z.-Y.D.); jgnlh@163.com (G.-N.J.); freeskywang@163.com (W.W.); sunyimin224@163.com (Y.-M.S.); chenweixunclark@163.com (W.-X.C.); chenlin_tj@126.com (L.C.); lianghuifang1997@126.com (H.-F.L.); 2 Department of Nephrology, Union Hospital, Tongji Medical College, Huazhong University of Science and Technology, Wuhan 430030, China; 3 Division of Hematology and Oncology, Department of Medicine, University of Alabama at Birmingham, Birmingham, AL 35294, USA; dattapk@uab.edu; 4 Division of Nephrology and Hypertension, Department of Medicine, Vanderbilt University, Nashville, TN 37235, USA; ming-zhi.zhang@vanderbilt.edu

**Keywords:** liver fibrosis, Activin A, oval cell, connective tissue growth factor, Smad proteins

## Abstract

Liver progenitor cells (LPCs) are activated in chronic liver damage and may contribute to liver fibrosis. Our previous investigation reported that LPCs produced connective tissue growth factor (CTGF/CCN2), an inducer of liver fibrosis, yet the regulatory mechanism of the production of CTGF/CCN2 in LPCs remains elusive. In this study, we report that Activin A is an inducer of CTGF/CCN2 in LPCs. Here we show that expression of both Activin A and CTGF/CCN2 were upregulated in the cirrhotic liver, and the expression of Activin A positively correlates with that of CTGF/CCN2 in liver tissues. We go on to show that Activin A induced *de novo* synthesis of CTGF/CCN2 in LPC cell lines LE/6 and WB-F344. Furthermore, Activin A contributed to autonomous production of CTGF/CCN2 in liver progenitor cells (LPCs) via activation of the Smad signaling pathway. Smad2, 3 and 4 were all required for this induction. Collectively, these results provide evidence for the fibrotic role of LPCs in the liver and suggest that the Activin A-Smad-CTGF/CCN2 signaling in LPCs may be a therapeutic target of liver fibrosis.

## 1. Introduction

Liver fibrosis is regarded as an inappropriate tissue repair response during chronic liver injury [1]. Persistent liver fibrosis is a major contributor to the development of cirrhosis that is associated with portal hypertension, end-stage liver disease and initiation of hepatocellular carcinoma (HCC) [2]. Understanding the mechanisms of liver fibrosis is essential to identify novel strategies for the presentation of cirrhosis. In the normal liver, liver regeneration is predominantly mediated by the activation and expansion of liver parenchymal cells, such as hepatocytes and cholangiocytes after liver injury. In the cirrhotic liver the capacity of expansion of liver parenchymal cells is limited by the disturbed tissue architecture. This is associated with the activation and expansion of liver progenitor cells (LPCs) the subsequently differentiate into both hepatocytes and cholaniocytes [3,4]. In morphology, LPCs (also termed as oval cells in rodents) are recognized as a population of cells that are located in the canals of Herring and are characterized by an ovoid nucleus and small size in comparison to differentiated hepatocytes [5]. Recent evidence has demonstrated that LPCs may contribute to liver fibrosis through the release of factors including connective tissue growth factor (CTGF/CCN2) that induce the production of an excessive extracellular matrix (ECM) [6,7]. However the mechanism by which LPC’s are stimulated to express these factors is not fully understood.

Activin A belongs to the Activin family, a major branch of the TGF-β superfamily. Activin A is formed through the homodimerization of two inhibin-βA subunits. Activin A activates canonical Smad signaling through initial interaction with activin type II receptors (ActRII and ActRIIB) and subsequent recruitment of the Activin receptor type I receptor (ActRIB): activin receptor-like kinase 4 (ALK4) [8]. In the liver, Activin A inhibits proliferation [9] and induces apoptosis [10] of hepatocytes and contributes to the termination of liver regeneration [11]. Our previous work demonstrated that the proliferation of LPCs and LPC-mediated liver regeneration was controlled by Activin A [12]. Previous studies have reported that the expression of Activin A is elevated in the fibrotic liver [13] and Activin A contributes to liver fibrosis through induction of fibrotic matricellular proteins such as CTGF/CCN2 in hepatocytes [14] and hepatic stellate cells (HStCs) [15,16].

CTGF/CCN2 is one of the most important fibrotic matricellular proteins and serves as a master switch in liver fibrosis [17]. Previous studies have demonstrated that LPCs are a source of CTGF/CCN2 production and secretion in the liver [6,18]. In addition to LPCs, hepatocytes, cholangiocytes and hepatic stellate cells (HSCs) can express CTGF/CCN2 in response to pro-fibrotic factors. The mechanisms by which CTGF/CCN2 is induced have been reported to be cell context dependent [19,20,21]. In hepatocytes, production of CTGF/CCN2 is modulated by transforming growth factor-β (TGF-β) and Activin A in the cell niche [17,22,23]. In liver progenitor cells (LPCs), our previous work found that TGF-β induces the production and secretion of CTGF/CCN2 [6], although the signaling pathways by which this occurs in LPCs was not explored.

Considering that LPCs are activated, and the expression of Activin A is up-regulated in the cirrhotic liver, we set out to determine the contribution of Activin A towards CTGF/CCN2 induction in LPCs together with the mechanism by which this occurs. We found that Activin A was an inducer of CTGF/CCN2 synthesis in LPCs, and this induction was mediated by Smad signaling. Our results elucidated that intracrine Activin A-ActRIB-Smad signaling was activated and contributed to the production of CTGF/CCN2 in LPCs.

## 2. Results

### 2.1. The Expression of Activin A and Connective Tissue Growth Factor (CTGF/CCN2) Are Elevated in the Cirrhotic Liver

**Figure 1:**
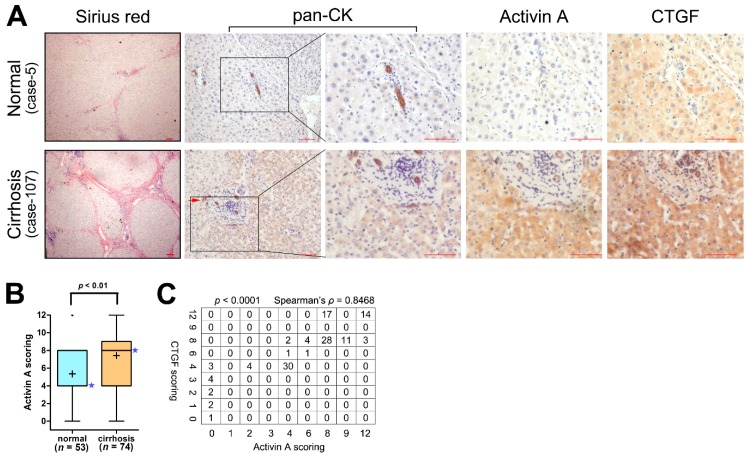
The expression of Activin A and connective tissue growth factor (CTGF/CCN2) were elevated in the cirrhotic liver. (**A**) Serial sections of human liver samples were subjected to Sirius red staining and immunohistochemical analysis of pan-cytokeratin, Activin A and CTGF/CCN2. Representative images were shown, LPC pools in the liver were indicated by the red arrow. The length of the scale bar in the lower right corner of every image is 100 μm; (**B**) Liver sections were divided into two groups: normal group and cirrhosis group according to the Histology Activity Index (HAI) score system. The immunohistochemistry score of Activin A in each immnuostained liver section was evaluated. The expression of Activin A in each group was shown, the pentagram indicated the median score of each group. Wilcoxon test, *p* < 0.01; (**C**) Correlation of expression levels of Activin A and CTGF/CCN2 in human liver samples. The Spearman’s ρ = 0.8468, *p* < 0.0001.

Our previous study confirmed that LPCs were activated and CTGF/CCN2 was up-regulated in the cirrhotic liver [6]. To explore the role of Activin A in LPCs during liver fibrosis, we first performed immunohistochemistry analyses to measure the expression of Activin A in normal and cirrhotic liver tissues. We found that increased Activin A levels in the cirrhotic group when comparing with that in the normal group (Figure 1A,B), and the expression of Activin A positively correlated with the production of CTGF/CCN2 in liver tissue (Figure 1C). We then measured pan-cytokeratin (pan-CK) in immunostained liver tissue as a marker of LPCs, and confirmed the existence of LPCs in the cirrhotic liver (Figure 1A).

### 2.2 Activin A Induces CTGF/CCN2 Synthesis in Liver Progenitor Cells (LPCs)

**Figure 2:**
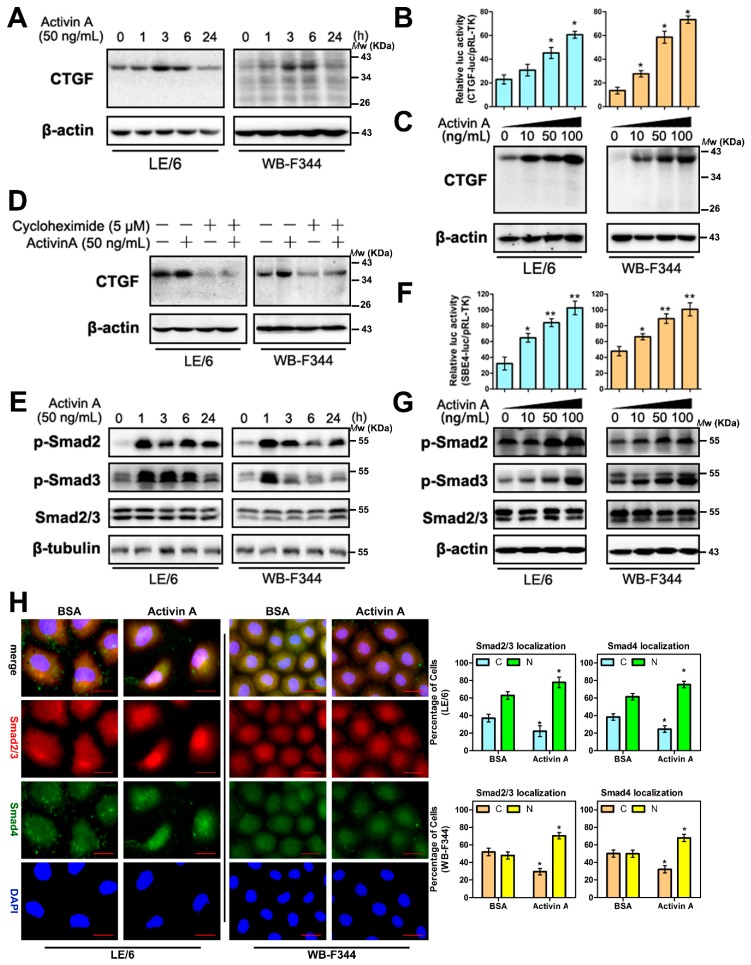
Activin A induces the production of CTGF/CCN2 in liver progenitor cells (LPCs). (**A**,**E**) LE/6 and WB-F344 cells were treated with Activin A (50 ng/mL) for the indicated times. Lysates were subjected to Western blot analyses with antibodies against indicated proteins. β-Actin (**A**) or β-tubulin (**E**) was used as a loading control; (**B**,**F**) LE/6 or WB-F344 cells was co-transfected with pRL-TK and CTGF-luc (**B**), or Smad binding elements (SBE4-luc) (**F**) plasmids as indicated, and then treated with Activin A at different concentrations as indicated for 16 h. Luciferase activity was normalized to renilla luciferase activity and expressed as the means ± S.E.M. of triplicate measurements. * *p* < 0.05 and ** *p* < 0.01 compared with the first bar; (**C**,**G**) LE/6 and WB-F344 cells were stimulated with Activin A at indicated concentrations. Lysates were subjected to Western blot analyses with antibodies against indicated proteins. β-Actin was used as loading control; (**D**) LE/6 and WB-F344 cells were treated with Activin A (50 ng/mL) and cycloheximide (5 μM) as indicated for 6 h. Lysates were subjected to Western blot analyses with antibodies against CTGF/CCN2. β-actin was used as a loading control; (**H**) **Upper panel**: LE/6 and WB-F344 cells were treated with Activin A (50 ng/mL) for 1 h and then subjected to double immunofluorescent staining of Smad2/3 (red) and Smad4 (green); DAPI were used to show the location of the nucleus (blue); scale bar, 20 μm; **Lower panel**: quantification of cells that showed Smad2/3 (**left**) and Smad4 (**right**) cytosolic (C) and/or nuclear (N) staining. The pink color of the merged image showed the merged staining of Smad2/3 (red), Smad4 (green), and DAPI (blue). * *p* < 0.05, compared with their control group (BSA), respectively.

A previous study demonstrated that Activin A is an upstream inducer of CTGF/CCN2 in hepatocytes [14]. To determine whether Activin A has a similar effect on LPCs, we used LE/6 and WB-F344 LPC cell lines to investigate functions of LPCs *in vitro* [6,24]. Western blotting and luciferase reporter assays of CTGF/CCN2 promoter showed that after treatment with Activin A, the production of CTGF/CCN2 in LPC cell lines was elevated in a dose and time dependent manner (Figure 2A–C). Treatment with cycloheximide blocked Activin A mediated CTGF/CCN2 production in LPCs, which suggested that Activin A caused *de novo* synthesis of CTGF/CCN2 in LPCs (Figure 2D). We next stimulated LPC’s with Activin A and measured Smad2 and Smad3 activation by Western immunoblotting. We found that Activin treatment resulted in dose and time dependent elevation of phosphorylated Smad2 and 3 (Figure 2E,G). Luciferase reporter assays revealed that the luciferase activity of Smad binding elements (SBE4-luc) were increased in transfected LPCs stimulated with Activin A (Figure 2F). Immunofluorescence revealed that Activin A induced Smad2, 3 and 4 translocation into the nucleus (Figure 2H). These results demonstrated that the Smad signaling was activated in response to Activin A in LPCs. In addition, treating LE/6 cells with inhibitor of receptor Activin-like kinase (ALK) 4 and 5 (SB431542) [25] suppressed Smad phosphorylation, whereas the specific ALK5 inhibitor (LY364947) had no effect on Activin A inducible phosphorylation of Smad2 (Figure S1B). Collectively, these results revealed that in LPCs, Activin A activated Smad signaling through ALK4, and Activin A mediated the production of CTGF/CCN2.

### 2.3. Intracrine Activin A Signaling Is Activated in LPCs

**Figure 3:**
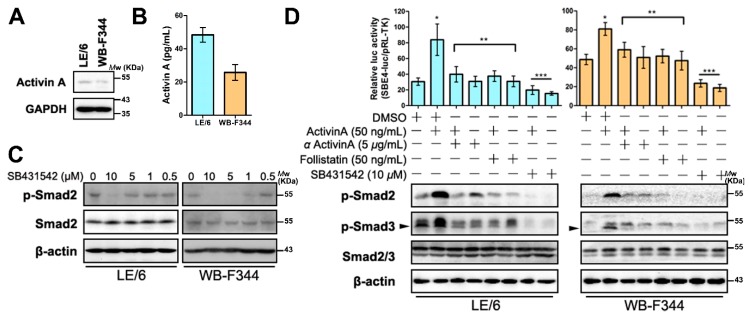

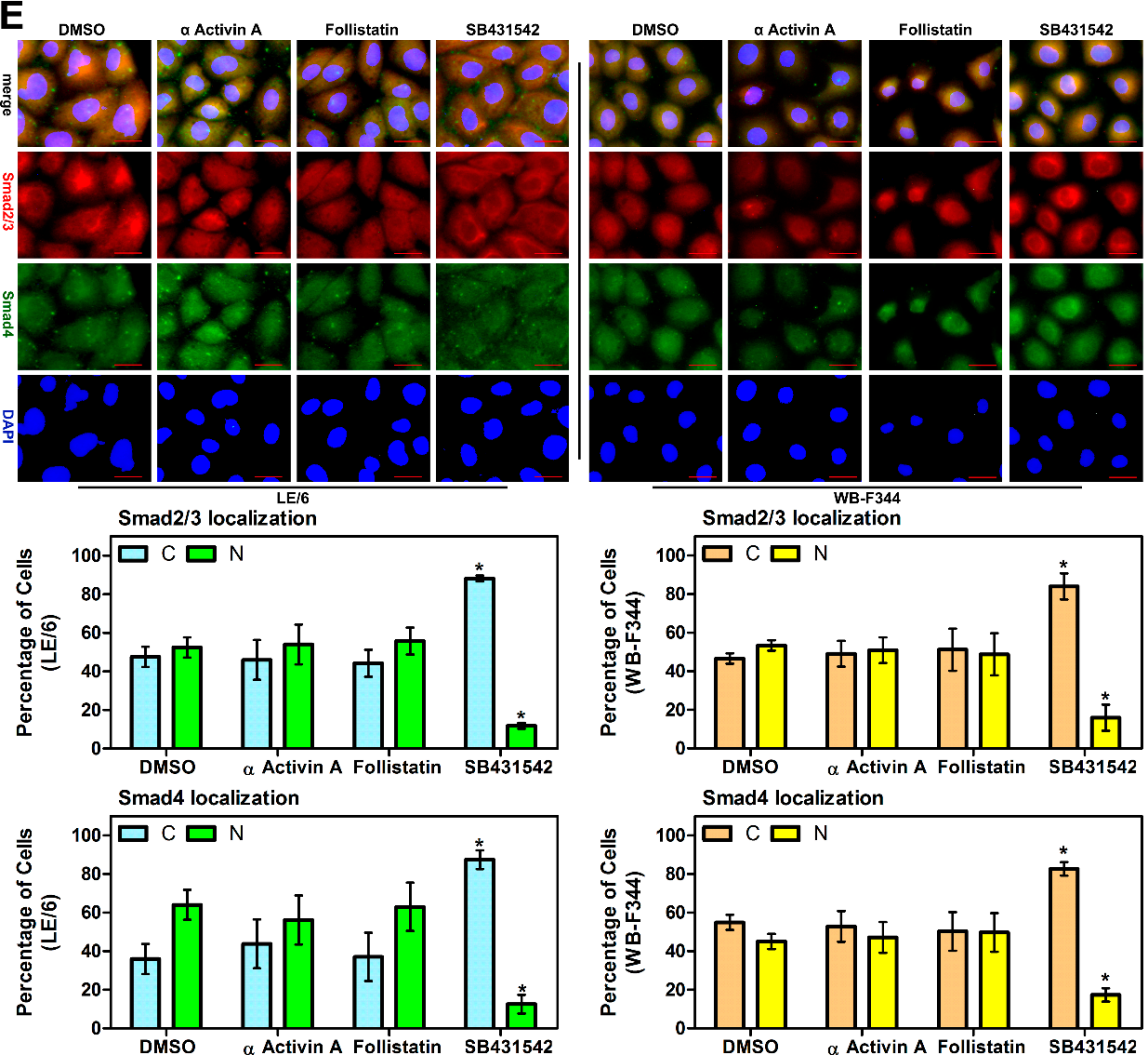
Intracrine Activin A signaling is activated in LPCs. (**A**) Cell lysates from LE/6 and WB-F344 cells were subjected to Western blot analyses with antibodies against Activin A. Glyceraldehyde-3-phosphate dehydrogenase (GAPDH) was used as a loading control; (**B**) Activin A secreted from WB-F344 and LE/6 cells was measured by enzyme-linked immunosorbent assay (ELISA). All results are means ± S.E.M. of triplicate measurements. Each experiment was repeated 3 times; (**C**) LE/6 and WB-F344 cells were treated with SB431542 at indicated concentrations. Cell lysates were subjected to Western blot analyses with antibodies against indicated proteins. β-Actin was used as loading control; (**D**) **Upper panel**: LE/6 or WB-F344 cells was co-transfected with pRL-TK and SBE4-luc plasmids and treated with indicated cytokines and inhibitors for 16 h. Luciferase activity was normalized to renilla luciferase activity and expressed as the means ± S.E.M. of triplicate measurements. Bars were compared as follows: * *p* < 0.01 compared with the first; ** *p* > 0.05 compared with the first and *p* < 0.05 compared with the second; *** *p* < 0.05 compared with the first and *p* < 0.01 compared with the second; **Lower panel**: LE/6 or WB-F344 cells was treated with indicated cytokines and inhibitors for indicated times. Lysates were subjected to Western blot analyses with antibodies against phospho-Smad2, phospho-Smad3 and Smad2/3. β-Actin was used as a loading control, arrow head indicated the band of p-Smad3; (**E**) LE/6 and WB-F344 cells were treated with indicated antibodies or inhibitors for 3 h and then subjected to double immunofluorescent staining of Smad2/3 (red) and Smad4 (green). DAPI were used to show the location of the nucleus (blue); scale bar, 20 μm (Upper panel). Quantification of cells that showed Smad2/3 and Smad4 cytosolic (C) and nuclear (N) staining (Lower panel). * *p* < 0.05, compared with their control group (DMSO), respectively.

We have previously demonstrated that serine residues in the carboxyl termini of Smad2 are autonomously phosphorylated by Western blot analyses, and we have proved the Smad2 and Smad3 are partly located in the nucleus of LPCs [6]. These results implied that Smad signaling was activated autonomously in LPCs. Considering that in LPCs, Smad signaling was activated by Activin A (Figure 2E,F), these results implied that the Activin A-Smad signaling may also be autonomously activated. To prove this hypothesis, we evaluated the expression of Activin A in LPC cell lines. Western blot and enzyme-linked immunosorbent assay (ELISA) analyses showed that Activin A was expressed and secreted in LPCs (Figure 3A,B). A previous study reported that Activin A signaling was activated by intracellular cytokine (intracrine signaling) in hepatocytes [14]. To test whether this phenomenon also existed in LPCs, we used inhibitors of Activin A signaling, as well as Activin A to treat LPC cell lines. We performed immunofluorescence, Western blot, and luciferase reporter assays. The results revealed that after cells were treated with Activin receptor kinase inhibitor (SB431542), which was able to inhibit extracellular and intracellular Activin A signaling [14], the phosphorylation of Smad2 and Smad3 in their carboxyl termini were both reduced (Figure 3C,D and S1B). Luciferase reporter analyses revealed that the SBE4-luc activity of LPCs with or without Activin A treatment were both decreased when compared with their control groups (Figure 3D). However, treating LPCs with neutralizing antibody (αActivin A) or antagonist (follistatin) of Activin A, which could inhibit extracellular Activin A signaling only, blocked exogenous Activin A stimulated Smad signaling but had no effect on intracrine Smad signaling (Figure 3D and S1A). Immunofluorescence analyses of LPCs showed that SB431542 was able to induce Smad2, 3 and 4 translocate to the cytosol, whereas αActivin A and follistatin could not (Figure 3E). Collectively, these results revealed that intracellular Activin A contributed to the autonomous Smad signaling in LPCs.

### 2.4. Knock Down of Activin A in LPCs Causes Reduced Activity of Smad Signaling

**Figure 4:**
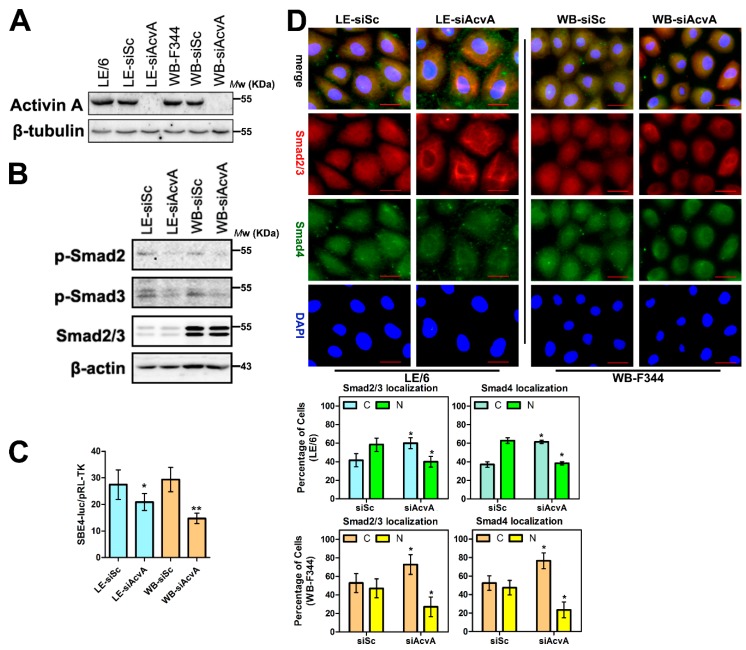
Knock down of Activin A in LPCs causes reduced activity of Smad signaling. (**A**,**B**) LE/6 and WB-F344 cells were transfected with siRNA duplexes against Activin A (WB-siAcvA or LE-siAcvA cells) or scramble siRNA (WB-siSc or LE-siSc cells), Lysates were subjected to Western blot analyses with antibodies against indicated proteins. β-Tubulin (**A**) or β-actin (**B**) was used as a loading control; (**C**) LE-siSc /LE-siAcvA or WB-siSc /WB-siAcvA cells were co-transfected with pRL-TK and SBE4-luc. Luciferase activity was normalized to renilla luciferase activity and expressed as the means ± S.E.M. of triplicate measurements. * *p* < 0.05, compared with their control group (siSc), respectively; and (**D**) **Upper panel**: LE-siSc /LE-siAcvA or WB-siSc /WB-siAcvA cells were subjected to double immunofluorescent staining of Smad2/3 (red) and Smad4 (green). DAPI were used to show the location of the nucleus (blue); scale bar, 20 μm; **Lower panel**: quantification of cells that showed Smad2/3 (**left**) and Smad4 (**right**) cytosolic (C) and/or nuclear (N) staining. * *p* < 0.05, compared with their control group (siSc), respectively.

Considering that SB431542 is a kinase inhibitor of both ALK4 and ALK5 [25] and our previous study showed that TGF-β-ALK5 signaling induces production of CTGF/CCN2 in LPCs [6], we next confirmed the contribution of intracellular Activin A signaling in LPCs. We knocked down Activin A in LPCs by specific siRNAs (Figure 4A) and performed Western blot, immunofluorescence and transcriptional response assays. After Activin A was knocked down, the phosphorylation of Smad2 and 3 in their carboxyl termini, and SBE4-luc luciferase activity were reduced (Figure 4B,C), and an increased proportion of Smad2, 3 and 4 was found in the cytosol of LPCs (Figure 4D).

### 2.5. Intracrine Activin A Signaling Contributes to the CTGF/CCN2 Production in LPCs

**Figure 5:**
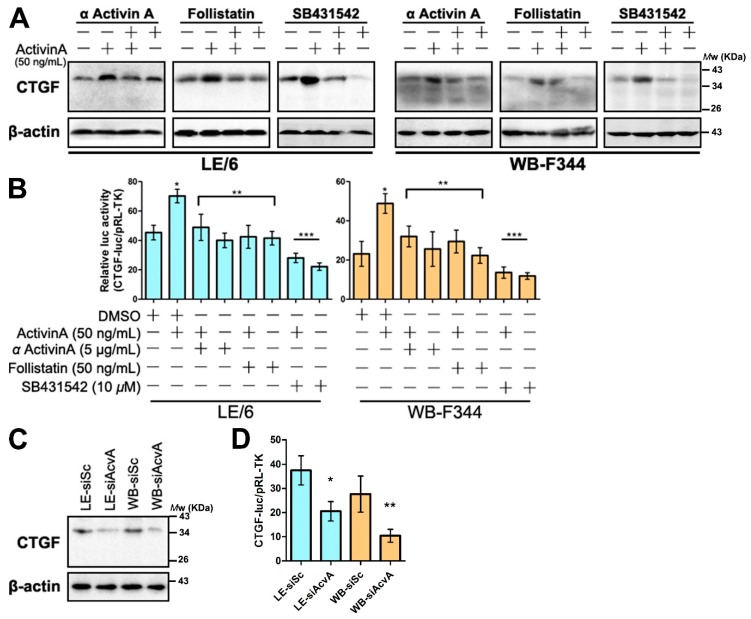
Activin A induced the production of CTGF/CCN2 in LPCs. (**A**) LE/6 or WB-F344 cells was treated with indicated cytokines and inhibitors for indicated times. Lysates were subjected to Western blot analyses to measure the expression of CTGF/CCN2. β-Actin was used as a loading control; (**B**) LE/6 or WB-F344 cells was co-transfected with pRL-TK and CTGF-luc plasmids and treated with indicated cytokines and inhibitors for 16 h. Luciferase activity was normalized to renilla luciferase activity and expressed as the means ± S.E.M. of triplicate measurements, * *p* < 0.01 compared with the first; ** *p* > 0.05 compared with the first and *p* < 0.05 compared with the second; *** *p* > 0.05 compared with the first and *p* < 0.01 compared with the second; (**C**) Lysates of LE-siSc/LE-siAcvA cells and WB-siSc/WB-siAcvA cells were subjected to Western blot analyses to measure the expression of CTGF/CCN2. β-Actin was used as a loading control; (**D**) LE-siSc /LE-siAcvA or WB-siSc/WB-siAcvA cells were co-transfected with pRL-TK and CTGF-luc. Luciferase activity was normalized to renilla luciferase activity and expressed as the means ± S.E.M. of triplicate measurements. * *p* < 0.05; and ** *p* < 0.01, compared with their control group (siSc), respectively.

We found that the autonomous production of CTGF/CCN2 existed in LPCs (Figure 2A–D). To test whether this production of CTGF/CCN2 in LPCs was modulated by intracrine Activin A signaling, we treated LPC cell lines with inhibitors mentioned previously. The results revealed that neutralizing antibody or antagonist of Activin A inhibited the exogenous Activin A induced CTGF/CCN2 production, whereas it was not able to inhibit the autonomous production of CTGF/CCN2. By contrast the use of the kinase inhibitor (SB431542) reduced the production of CTGF/CCN2 via both exogenous and endogenous signals (Figure 5A,B). Moreover, Western blot and CTGF-luc reporter analyses showed that the production of CTGF/CCN2 was reduced after Activin A was knocked down in LPCs (Figure 5C,D). These results further confirmed that intracellular Activin A signaling contributed to the synthesis of CTGF/CCN2 in LPCs.

### 2.6. Activin A Mediated the Production of CTGF/CCN2 through Smad Signaling in LPCs

**Figure 6:**
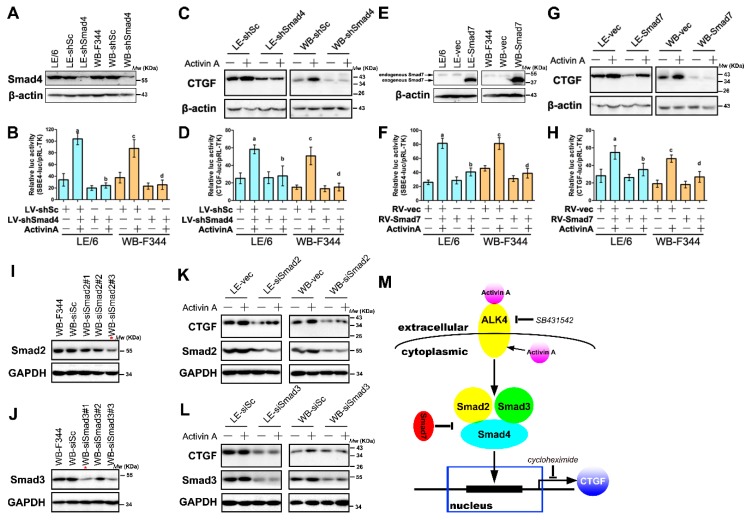
Activin A induced the production of CTGF/CCN2 through Smad signaling in LPCs. (**A**,**E**) LE/6 and WB-F344 cells were transfected with retrovirus carrying shRNA against Smad4 (WB-shSmad4 or LE-shSmad4 cells) or scramble shRNA (WB-shSc or LE-shSc cells) (**A**), or ectopic expressed Smad7 (WB-Smad7 and LE-Smad7 cells) or vector (WB-vec and LE-vec cells) by retrovirus (**E**), Western blot analyses showed the expression of Smad4 (**A**) and Smad7 (**E**). β-Actin was used as a loading control; (**B**,**D**,**F**,**H**) WB-shSmad4/LE-shSmad4 and WB-shSc/LE-shSc cells (**B** and **D**); or WB-Smad7/LE-Smad7 cells and WB-vec/LE-vec cells (**F** and **H**) were co-transfected with pRL-TK and SBE4-luc (**B** and **F**), or CTGF-luc (**D** and **H**). Luciferase activity was normalized to renilla luciferase activity and expressed as the means ± S.E.M. of triplicate measurements; the bars were compared as follows: a, *p* < 0.05, second with first; b, *p* < 0.05, fourth with second; c, *p* < 0.05, sixth with fifth; d, *p* < 0.05, eighth with sixth); (**C**,**G**) WB-shSmad4/LE-shSmad4 and WB-shSc/LE-shSc cells (**C**); or WB-Smad7/LE-Smad7 cells and WB-vec/LE-vec cells (**G**) were treated with Activin A (50 ng/mL) for 6 h. Lysates were subjected to Western blot analyses with antibodies against CTGF/CCN2. β-Actin was used as a loading control; (**I**,**J**) WB-F344 cells were transfected with siRNA duplexes targeting rat Smad2 (**I**), or Smad3 (**J**), or scrambled siRNA, and cell lysates were subjected to immunoblotting assay with antibody against Smad2 (**I**) or Smad3 (**J**). GAPDH was used as a loading control. *, siRNA duplexes with the highest knock-down efficiency was selected and used in the following experiments; (**K**,**L**) WB-F344 and LE/6 cells were transfected with validated specific siRNAs against Smad2 (siSmad2 cells) (**K**), or Smad3 (siSmad3 cells) (**L**), or scramble shRNA (siSc cells) and transfected cells were treated with Activin A (50 ng/mL) for 6 h. Lysates were subjected to Western blot analyses with antibodies against CTGF/CCN2, and Smad2 (**K**) or Smad3 (**L**). GAPDH was used as a loading control; (**M**) Schematic illustrations of the Activin A signaling in the expression of CTGF/CCN2 in LPCs. Exogenous and endogenous Activin A activates and receptor I (ActRIB, ALK4) (↓), leading to the phosphorylation and activation of Smad2 and Smad3 (↓), which form a complex with Smad4 to induce the *de novo* synthesis of CTGF/CCN2 (↓). Both Smad2 and Smad3-regulated signaling contribute to the induction of CTGF/CCN2 in LPCs, and this induction can be inhibited by SB431542 (┴), an ALK4 inhibitor, and inhibitory Smads such as Smad7 (┴); ↓,activation; ┴, inhibition.

Previous studies proved that Activin A mediated CTGF/CCN2 production via Smad signaling in hepatocytes [14]. To investigate whether Activin A-Smad signaling induces the production of CTGF/CCN2 in LPCs, we first knocked down Smad4, a crucial component of Smad signaling, by lentivirus in WB-F344 and LE/6 cells (Figure 6A). Luciferase reporter analyses revealed that the activity of SBE4-luc in transfected LPC cell lines was reduced and was not responsive to the stimulation of Activin A (Figure 6B). This data revealed that the Activin A-Smad signaling was abrogated after knock down of Smad4 in LPCs. Moreover, Western blot and luciferase activity analyses revealed that knock down of Smad4 significantly reduced Activin A inducible production of CTGF/CCN2 in LPCs (Figure 6C,D). In addition, overexpression of Smad7 (Figure 6E), a member of inhibitory Smads, suppressed Activin A-Smad signaling (Figure 6F), and reduced Activin A stimulated CTGF/CCN2 expression in LPCs (Figure 6G,H).

Previous studies reported that Smad2 and 3 play distinct roles in the induction of CTGF/CCN2 by TGF-β in hepatocytes [19,20]. To investigate the precise role of Smad2 and Smad3 in the production of CTGF/CCN2 by Activin A-Smad signaling in LPCs, we firstly knocked down the expression of Smad2 and Smad3 respectively in LPCs by using specific siRNA duplexes. We tested three pairs of siRNA duplexes, and the one with the highest knockdown efficiency was selected (Figure 6I,J). The results of Western blot analyses revealed that in the presence of Activin A, knockdown of Smad2 (Figure 6K), or Smad3 (Figure 6L), caused decreased CTGF/CCN2 production in LPCs. Taken together, these results proved that Activin A inducible CTGF/CCN2 production in LPCs is mediated by Smad signaling, and Smad2, 3 and 4 were all necessary for this induction (Figure 6M).

## 3. Discussion

LPCs are activated and expanded during chronic liver damage and were initially found to contribute to tissue repair of the liver [4,26]. However, work by several groups has revealed that LPCs contribute to the process of liver fibrosis [7,27], though their precise roles have remained elusive. Our previous study reported that LPCs express and secrete CTGF/CCN2, a fibrogenic master switch, and this secretion was stimulated by TGF-β [6]. However, CTGF/CCN2 production in LPCs may be regulated by profibrogenic cytokines other than TGF-β. In this study, we confirmed that the expression of Activin A was increased in the cirrhotic liver, and demonstrated that Activin A was another inducer of CTGF/CCN2 in LPCs. We demonstrated that Activin A induces the production of CTGF/CCN2 through direct activation of Smad in LPCs. Collectively, these results suggested that Activin A exerted its fibrotic role through the induction of CTGF/CCN2 in LPCs, and our results provided new evidence in the relationship between LPCs and liver fibrosis.

Activin A is produced and secreted by various types of cells in the liver including hepatocytes [14], HSCs [28], cholangiocytes [29] and hepatoma cells [30]. Activin A is able to induce apoptosis of hepatocytes [10] and contributes to the production of ECM and fibrotic cytokines in HSCs and hepatocytes [14,15]. Inhibitors of Activin A have been proposed as therapies for liver fibrosis. Specifically, a previous study demonstrated that follistatin, a natural antagonist of Activin A, attenuated the progression of liver fibrosis via controlling the Activin A inducible HSC activation and hepatocyte apoptosis [28]. In this study, we found that Activin A was expressed in LPCs and Activin A induced expression of CTGF/CCN2, a fibrotic matricellular protein. Thus, the antifibrotic effects of follistatin may occur through targeting LPCs in the fibrotic liver. We found that in LPCs, both extracellular and intracellular Activin A contributed to the induction of CTGF/CCN2. Our *in vitro* experiments revealed that follistatin could only attenuate the induction of CTGF/CCN2 by exogenous Activin A but had no effect on intracrine Activin A signaling in LPCs. These results suggested that intracrine Activin A contributed to the permanent fibrotic signaling in LPCs and the existence of intracrine Activin A signaling could potentially limit the efficacy of several antifibrotic drugs that only inhibit fibrotic signaling activated by extracellular Activin A.

In this study, we found that Activin A mediated the production of CTGF/CCN2 in LPCs through Smad signaling; this induction and its related mechanism was similar with that in hepatocytes [14]. These results suggested that LPCs showed some characteristics of hepatocytes. A series of recent investigations confirm the plasticity of LPC pools that both the differentiation of LPCs into hepatocytes [31] and biliary cells and the dedifferentiation of hepatocytes [32], biliary cells [33,34] and HSCs [35] to LPCs exists in the liver [36]. Thus, though LPCs, hepatocytes, biliary cells and HSCs are different types, they may show several similar signaling cascades and related downstream effects. However, the sensitivity of LPCs and hepatocytes in responding to the similar signaling may different. For instance, TGF-β induces growth inhibition through Smad signaling in both hepatocytes and LPCs, whereas LPCs showed less sensitivity to the stimulation of TGF-β than that in hepatocytes due to glycosylation of the TGF-β receptor type II (TβRII) as well as high expression of Smad6 [37]. This reduced sensitivity may help LPCs maintain a growth advantage in the context of liver fibrosis.

The mechanism of CTGF/CCN2 induction via Activin A-Smad signaling was different from that of TGF-β signaling in LPCs, for which the induction of CTGF/CCN2 was predominantly through TGF-β activating mitogen-activated protein kinase (MAPK) signaling [6]. In addition, our previous study revealed that Activin A was not able to activate Smad-independent MAPK signaling in LPCs [12]. Though both TGF-β and Activin A are members of TGF-β superfamily, the diversity of downstream signaling cascades and related effects activated by TGF-β and Activin A in the same cell is not rare [38,39]. Collectively, our results revealed that both Smad signaling and Smad-independent signaling showed effects on the induction of CTGF/CCN2 in LPCs, and targeting these signaling in LPCs might have antifibrotic effects.

Previous studies demonstrated that in addition to promoting liver fibrosis, CTGF/CCN2 was critical for the expansion of LPCs during LPC-mediated liver regeneration [18,40,41]. In this study, we found that Activin A induced production of CTGF/CCN2 in LPCs. These results suggest that Activin A may show stimulatory effect on the expansion of LPCs through CTGF/CCN2. Our previous study reported that Activin A inhibited the proliferation of LPCs and contributed to the termination of LPC-mediated liver regeneration [12]. Collectively, our results suggest that Activin A shows both stimulatory and inhibitory effects on the activation and expansion of LPCs. Expansion of LPCs in the regenerating liver is modulated by complicated signaling networks [4]. Our results further imply that Activin A may cooperate with other signaling pathways to regulate the activation and expansion of LPCs, and may play distinct roles in LPC-mediated liver regeneration and fibrosis.

LPCs were demonstrated to be tumorigenic under pathological microenvironments such as cirrhosis [42,43]. Consistently, previous studies reported that high serum Activin A levels were found in HCC patients with cirrhosis [44,45]. A previous investigation demonstrated that the malignant transformation of LPCs occurred under the continuous activation of TGF-β-Smad signaling [43]. Whether Activin A-Smad signaling had similar oncogenic effects on LPCs need further exploration. In addition, CTGF/CCN2 was proven to have an oncogenic role in HCC; it contributes to tumor-stromal crosstalk [46], and promotes tumor cell growth, dedifferentiation, resistance to doxorubicin, and expression of inflammation-related proteins that contribute to carcinogenesis [47]. Thus, whether CTGF/CCN2 contributes to the malignant transformation of LPCs and hepatocarcinogenesis is worth further investigation.

## 4. Materials and Methods

### 4.1. Reagents

Cell culture medium, puromycin, and polybrene were purchased or obtained as described previously [6]. Cytokines and kinase inhibitors are listed in Table 1. Antibodies are listed in Table 2.

### 4.2. Liver Samples

One hundred and twenty-seven human liver samples used in this study were collected, and detailed clinicopathologic information was as described previously [6]. Sirius staining of liver tissues and measuring the severity of liver fibrosis were performed as described previously [6]. This study was conducted according to the Declaration of Helsinki principles and the procedure of human sample collection was approved by the Ethics Committee of Tongji Hospital, Tongji Medical College, Huazhong University of Science and Technology (Approval Number: TJ-C20140713, date of approval: 24 July 2014).

### 4.3. Immunohistochemistry and Double Immunofluorescent Analyses

Immunohistochemistry of paraffin-embedded liver slices and double immunofluorescence analyses of cells were carried out as described previously [6]. Immunohistochemical staining score of each slice and counting cells in each slice were performed as described previously [6].

### 4.4. Cell Lines and Cell Culture

LPC cell lines LE/6 and WB-F344, the lentivirus packaging cell line 293T, and the retrovirus packaging cell line RetroPack PT67 were cultured as described previously [6,48]. Cells were incubated overnight in culture medium without serum before used in following experiments.

### 4.5. Plasmids

pBabe-puro, pBabe-Flag-Smad7, SBE4-luc, pRL-TK, pHelper 1.0, pHelper 2.0, CTGF-luc, and pSCSIL001-shSmad4 were purchased or generated as described previously [6].

### 4.6. Virus Production, Cells Infection and Selection of Stable Cell Clones

Production and concentration of retroviral (RV) or lentiviral (LV) supernatants were performed as described previously [49]. Infection of LPC cell lines by retrovirus or lentivirus were performed as described previously [6].

### 4.7. Transient RNA Interference

Transfection of the small interfering RNA (siRNA) duplexes were carried out as described previously [6]. Scrambled siRNA and siRNA targeting rat *inhibin-βA* sequence were designed and validated as described previously [50]. siRNA duplexes targeting rat *smad2* and *smad3* mRNA and scrambled siRNA were designed and validated by Ribobio (Guangzhou, China). All siRNAs used in this study were synthesized by Ribobio. For knock down of Activin A (homodimer of *inhibin-βA*) in LPC cells, a mixture of 4 validated different siRNA duplexes were used for transfection of cells.

### 4.8. Luciferase Reporter Analyses

Luciferase reported analyses of SBE4-luc or CTGF-luc report were carried out as described in the previous study [6].

### 4.9. Western Blot Analyses

Western blot analyses were performed as described previously [49].

### 4.10. Enzyme-Linked Immunosorbent Assay (ELISA) for Activin A

5 × 10^5^ cells were seeded in each well of 6-well plates and incubated for 24 h in serum free medium. Cell counting was performed by using Cellometer Mini (Nexcelom Bioscience, Massachusetts, USA) according to instructions of manufacturer’s. Supernatants were collected for a rat Activin A assay following the manufacturer’s instructions (R & D System, MN, USA).

### 4.11. Statistical Analyses

Experimental data are presented as mean ± SEM of three independent experiments. Statistical analyses were carried out by Student’s *t*-test, analysis of variance (ANOVA) plus Bonferroni *post hoc* test, Wilcoxon test, or Spearman’s rank correlation coefficient as appropriate. *p* < 0.05 was considered statistically significant.

## 5. Conclusions

In conclusion, our study demonstrates that Activin A mediates the *de novo* synthesis of CTGF/CCN2 in LPCs. In addition, we found that Activin A signaling was intracellular activated, and intracrine Activin A signaling also contributes to the synthesis of CTGF/CCN2 in LPCs. Moreover, we demonstrated Activin A-inducible CTGF/CCN2 synthesis in LPCs via Smad signaling, and that Smad2, 3 and 4 collaboratively contributed to this induction. Our investigation provides molecular evidence for the fibrotic role of LPCs in the liver and suggests that the Activin A-Smad-CTGF/CCN2 axis in LPCs may be a potential therapeutic target of liver fibrosis.

## Figures and Tables

**Table 1:** Cytokines, kinase inhibitors and other chemicals used in this study.

Chemicals	Manufacturers
Recombinant human Activin A	120-14E, PeproTech, Rocky Hill, NJ, USA
Recombinant human Follistatin 315 aa 30-344	4889-FN/CF, R&D Systems, Minneapolis, MN, USA
SB431542	301836-41-9, Cayman, Ann Arbor, MI, USA
LY364947	616451, Merck Calbiochem, Darmstadt, Germany
Cycloheximide	SI005, Beyotime Institute of Biotechnology, Haimen, Jiangsu, China

**Table 2:** Antibodies used in this study.

Antigens	Manufacturers	Application
CTGF/CCN2	sc-14939, Santa Cruz Biotechnology, Santa Cruz, CA, USA	1:200 for WB, I:50 for IHC
Pan-cytokeratin	IR053, Dako, Glostrup, Denmark.	Ready-to-Use for IHC
Activin A	AF338, R&D Systems, Minneapolis, MN, USA	1:50 (5 μg/mL) for IHC
Activin A	MAB3381, R&D Systems, Minneapolis, MN, USA	neutralization
Activin A	5624-1, Epitomics, Burlingame, CA, USA	1:500 for WB
Phospho-Smad2 (Ser465/467)	#3108, Cell Signaling Technology, Beverly, MA, USA	1:2000 for WB
Phospho-Smad3 (Ser423/425)	1880-1, Epitomics, Burlingame, CA, USA	1:2000 for WB
Smad2/3	sc-133098, Santa Cruz Biotechnology, Santa Cruz, CA, USA	1:500 for WB, 1:50 for IF
Smad4	1676-1, Epitomics, Burlingame, CA, USA	1:2000 for WB, 1:100 for IF
Smad7	3894-1, Epitomics, Burlingame, CA, USA	1:1000 for WB
β-Actin	sc-47778, Santa Cruz Biotechnology, Santa Cruz, CA, USA	1:10000 for WB
β-Tubulin	M30109, Abmart, Shanghai, China.	1:5000 for WB
GAPDH	KC-5G4, KangChen Bio-tech, Shanghai, China.	1:20000 for WB
Alexa Flour 488-conjugated anti-rabbit IgG	Beyotime Institute of Biotechnology, Haimen, Jiangsu, China	1:500 for IF
Alexa Flour 555-conjugated anti-mouse IgG	Beyotime Institute of Biotechnology, Haimen, Jiangsu, China	1:500 for IF
Horseradish peroxidase (HRP) conjugated anti-rabbit IgG	Jackson ImmunoResearch Laboratories, Inc. West Grove, PA, USA	1:5000 for WB
HRP conjugated anti-mouse IgG	Jackson ImmunoResearch Laboratories, Inc. West Grove, PA, USA	1:5000 for WB
HRP conjugated anti-goat IgG	Jackson ImmunoResearch Laboratories, Inc. West Grove, PA, USA	1:5000 for WB

IHC: Immnuohistochemistry; IF: Immnuoflurorescence; WB: Western Blot; CTGF/CCN2: Connective tissue growth factor.

## Supplementary Materials

Supplementary materials can be found at http://www.mdpi.com/1422-0067/17/3/408/s1.

## Abbreviations

CTGF/CCN2: Connective tissue growth factor
LPCs: Liver progenitor cells
HCC: Hepatocellular carcinoma
CHX: Cycloheximide
shRNA: Small hairpin RNA
ALK4: Activin receptor-like kinase 4
HSCs: Hepatic stellate cells
TGF-β: Transforming growth factor β
TβRII: TGF-β receptor type II
RV: Retrovirus
LV: Lentivirus
