# Supplementary material for: Activin A-Smad Signaling Mediates Connective Tissue Growth Factor Synthesis in Liver Progenitor Cells
Source: Int J Mol Sci. 2016 Mar 22;17(3):408. doi: 10.3390/ijms17030408 (PMC4813263; doi:10.3390/ijms17030408)
Supplement: Supplementary file 1 [file ijms-17-00408-s001.pdf]

# Supplementary Materials: Activin A-Smad Signaling Mediates Connective Tissue Growth Factor Synthesis in Liver Progenitor Cells

**Ze-Yang Ding, Guan-Nan Jin, Wei Wang, Yi-Min Sun, Wei-Xun Chen, Lin Chen, Hui-Fang Liang, Pran K. Datta, Ming-Zhi Zhang, Bixiang Zhang and Xiao-Ping Chen**

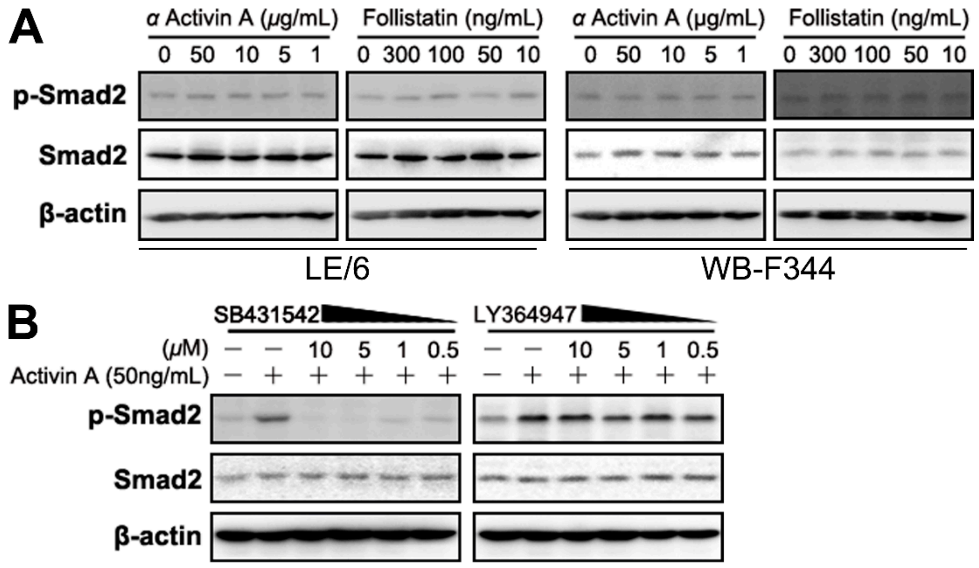


**Figure S1.** (**A**) LE/6 and WB-F344 cells were treated with antibody against Activin A, or follistatin at indicated concentrations. Cell lysates were subjected to Western blot analyses with antibodies against phospho-Smad2 and Smad2. β-actin was used as loading control; (**B**) LE/6 or WB-F344 cells was treated with indicated cytokines and inhibitors for 1 hour. Lysates were subjected to Western blot analyses to measure the expression of phospho-Smad2 and total Smad2. β-actin was used as a loading control.
